# Simultaneous changes in seed size, oil content and protein content driven by selection of *SWEET* homologues during soybean domestication

**DOI:** 10.1093/nsr/nwaa110

**Published:** 2020-05-27

**Authors:** Shoudong Wang, Shulin Liu, Jie Wang, Kengo Yokosho, Bin Zhou, Ya-Chi Yu, Zhi Liu, Wolf B Frommer, Jian Feng Ma, Li-Qing Chen, Yuefeng Guan, Huixia Shou, Zhixi Tian

**Affiliations:** State Key Laboratory of Plant Physiology and Biochemistry, College of Life sciences, Zhejiang University, Hangzhou 310058, China; State Key Laboratory of Plant Cell and Chromosome Engineering, Institute of Genetics and Developmental Biology, Innovation Academy for Seed Design, Chinese Academy of Sciences, Beijing 100101, China; University of Chinese Academy of Sciences, Beijing 100049, China; College of Resources and Environment, Fujian Provincial Key Laboratory of Haixia Applied Plant Systems Biology, Fujian Agriculture and Forestry University, Fuzhou 350002, China; Institute of Plant Science and Resources, Okayama University, Kurashiki 710-0046, Japan; Institute of Crop Science, Anhui Academy of Agricultural Sciences, Hefei 230031, China; Department of Plant Biology, School of Integrative Biology, University of Illinois at Urbana-Champaign, Urbana, IL 61801, USA; State Key Laboratory of Plant Cell and Chromosome Engineering, Institute of Genetics and Developmental Biology, Innovation Academy for Seed Design, Chinese Academy of Sciences, Beijing 100101, China; University of Chinese Academy of Sciences, Beijing 100049, China; Institute for Molecular Physiology and Cluster of Excellence on Plant Sciences (CEPLAS), Heinrich Heine University of Düsseldorf, Düsseldorf, Germany; Institute of Plant Science and Resources, Okayama University, Kurashiki 710-0046, Japan; Department of Plant Biology, School of Integrative Biology, University of Illinois at Urbana-Champaign, Urbana, IL 61801, USA; FAFU-UCR Joint Center for Horticultural Plant Biology and Metabolomics, Haixia Institute of Science and Technology, Fujian Agriculture and Forestry University, Fuzhou 350002, China; State Key Laboratory of Plant Physiology and Biochemistry, College of Life sciences, Zhejiang University, Hangzhou 310058, China; State Key Laboratory of Plant Cell and Chromosome Engineering, Institute of Genetics and Developmental Biology, Innovation Academy for Seed Design, Chinese Academy of Sciences, Beijing 100101, China; University of Chinese Academy of Sciences, Beijing 100049, China

**Keywords:** soybean, domestication, yield, seed quality, SWEET

## Abstract

Soybean accounts for more than half of the global production of oilseed and more than a quarter of the protein used globally for human food and animal feed. Soybean domestication involved parallel increases in seed size and oil content, and a concomitant decrease in protein content. However, science has not yet discovered whether these effects were due to selective pressure on a single gene or multiple genes. Here, re-sequencing data from >800 genotypes revealed a strong selection during soybean domestication on *GmSWEET10a*. The selection of *GmSWEET10a* conferred simultaneous increases in soybean-seed size and oil content as well as a reduction in the protein content. The result was validated using both near-isogenic lines carrying substitution of haplotype chromosomal segments and transgenic soybeans. Moreover, *GmSWEET10b* was found to be functionally redundant with its homologue *GmSWEET10a* and to be undergoing selection in current breeding, leading the the elite allele *GmSWEET10b*, a potential target for present-day soybean breeding. Both GmSWEET10a and GmSWEET10b were shown to transport sucrose and hexose, contributing to sugar allocation from seed coat to embryo, which consequently determines oil and protein contents and seed size in soybean. We conclude that past selection of optimal *GmSWEET10a* alleles drove the initial domestication of multiple soybean-seed traits and that targeted selection of the elite allele *GmSWEET10b* may further improve the yield and seed quality of modern soybean cultivars.

## INTRODUCTION

Studies have suggested that global agricultural production needs to be doubled by 2050 to meet the rapidly growing population and diet shifts [1–3], translating into a need for increasing crop production by 2.4% per year [4]. Soybean is a major, multiuse crop that globally makes up 56% of the oilseed production and >25% of the protein used in human food and animal feed [5]. At the current average rate of annual-yield increase, only 55% of the necessary increase in soybean production can be reached by 2050. Thus, breeding soybeans with higher yields is urgently needed [4].

Cultivated soybean (*Glycine max* [L.] Merr.) was domesticated from wild soybean (*G. soja* Sieb. & Zucc.) in China over a period of 5000 years [6]. Seeds of wild soybeans are generally smaller and contain higher levels of protein. Cultivated soybeans produce larger seeds with higher oil content (Supplementary Fig. 1). Thus far, it has not been reported that a single gene can simultaneously alter seed size, oil content and protein content, although a number of quantitative trait loci (QTLs) that govern seed size, oil content and protein content in soybean were identified through previous genetic analyses (SoyBase, https://soybase.org/). Therefore, it remains unclear whether the improvement in these traits was achieved by selection of a gene with pleiotropic effects on these traits or by selection of individual genes that only affect each trait.

Sucrose is the main source of carbon energy delivered via the phloem to developing seeds [7] and sugars derived from sucrose metabolism play pivotal roles in seed development for many species [7–13]. Previous studies have demonstrated that SWEET proteins play important roles in sugar translocation to seeds and consequently affect seed setting, filling and composition [14–18]. For example, in *Arabidopsis thaliana*, mutation of *AtSWEET11*/*12*/*15* impairs sucrose delivery from seed coat and endosperm to embryo and results in severe seed defects [16]. Similarly, knockout of *OsSWEET11* and *OsSWEET 15* in rice results in a complete loss of endosperm development [14,19]. Our previous study also illustrated that knockout of both *GmSWEET15a* and *GmSWEET15b* in soybean causes an extremely high rate of seed abortion [20].

In this study, we found that a pair of *SWEET* homologues, *GmSWEET10a* and *GmSWEET10b*, underwent stepwise selection that simultaneously altered seed size, oil content and protein content during soybean domestication. Our findings provide practical insights into how to improve soybean-seed traits, in particular seed size and oil content, by optimizing the combination of *GmSWEET10a* and/or *GmSWEET10b* alleles.

## RESULTS

### *GmSWEET10a* underwent selection during soybean domestication

**Figure 1.**
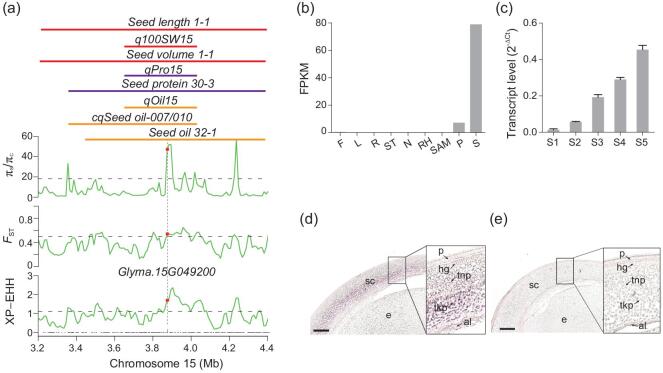
*GmSWEET10a* was identified as a candidate pleiotropic gene that influences seed size, fatty-acid content and protein content. (a) Genetic variations (π, *F*_ST_ and XP-EHH values) were calculated between *G. soja* (S) and the cultivar (C) across the 1.2-Mb genomic region of the *GmSWEET10a* locus. The dashed horizontal lines indicate the genome-wide thresholds (top 5% of the genome) of the selection signals. The solid lines above the plot represent genomic locations of QTLs retrieved from SoyBase (https://soybase.org/; Supplementary Table 1). The red, orange and purple lines are QTLs for the seed size, seed oil and protein contents, respectively. The black dashed lines above the *x*-axis are annotated genes in this region. The red dots denote the *GmSWEET10a* gene, i.e. *Glyma.15G049200*. (b) Expression pattern of *GmSWEET10a* in different organs in *Glycine max* (Gm). Expression values were obtained from Phytozome 12 (https://phytozome.jgi.doe.gov/pz/portal.html#). F, flower; L, leaf; R, root; ST, stem; N, nodule; RH, root hair; SAM, shoot apical meristem; P, pod; S, seed; FPKM, fragments per kilobase of exon per million mapped. (c) Transcript abundance of *GmSWEET10a* in seed coats at different stages. The expression was detected by reverse transcriptase quantitative polymerase chain reaction (RT-qPCR). Transcript levels were calculated relative to soybean cyclophilin 2 (*GmCYP2*). (d) and (e) RNA *in situ* hybridization of *GmSWEET10a* showing specific expression in the seed coats. Cross-sections of developing seeds at S2–S3 hybridized with antisense (d) or sense (e) probes for *GmSWEET10a.* sc, seed coat; e, embryo; p, palisade layer; hg, hourglass; tnp, thin-walled parenchyma; tkp, thick-walled parenchyma; al, aleurone layer. Scale bars, 200 μm.

Using whole-genome re-sequencing data from >800 accessions with an average coverage depth of >13X for each accession [21,22], we identified a selective sweep on chromosome 15 from 3.87 to 4.0 Mb. The fixation of this enlargement was observed by different methods, including calculating the nucleotide diversity (π), the fixation index (*F*_ST_) and the cross-population extended haplotype homozygosity (XP-EHH) (Fig. 1a). This selective sweep overlapped with several reported QTLs that related to seed size, oil content and protein content [23–28] (Fig. 1a and Supplementary Table 1). The results indicated that selected gene(s) in this region may be responsible for the simultaneous alternation of seed size, oil content and protein content in soybean domestication.

This selective sweep included 18 gene orders, among which *Glyma.15G049200* (previously named *GmSWEET10a* [20]) encoded a member of the SWEET family of sugar transporters (Fig. 1a). SWEET proteins play important roles in seed development [14–16,19,20]. Transcriptional profiling data from Phytozome 12 (https://phytozome.jgi.doe.gov/pz/portal.html#) showed that *GmSWEET10a* was specifically expressed in seed and pod (Fig. 1b). Transcriptome data from Gene Networks in Seed Development (http://seedgenenetwork.net/soybean) indicated that *GmSWEET10a* was mainly expressed in the seed coat (Supplementary Table 2). Quantitative RT-PCR (qRT-PCR) showed that the expression of *GmSWEET10a* in the seed coats progressively increased during seed development and reached their peaks at the full-seed stage (S5 stage in Supplementary Fig. 2 and Fig. 1c). *In situ* RNA hybridization confirmed that *GmSWEET10a* was preferentially expressed in the thick-walled parenchyma of the seed coat (Fig. 1d and e), which are important for sucrose translocation to the embryo [29–31]. The known functions of SWEET proteins and the expression pattern of *GmSWEET10a* indicated that it might be the gene responsible for the simultaneous alternation of seed size, oil content and protein content during soybean domestication.

### Association between seed traits and genetic variation of *GmSWEET10a*

**Figure 2.**
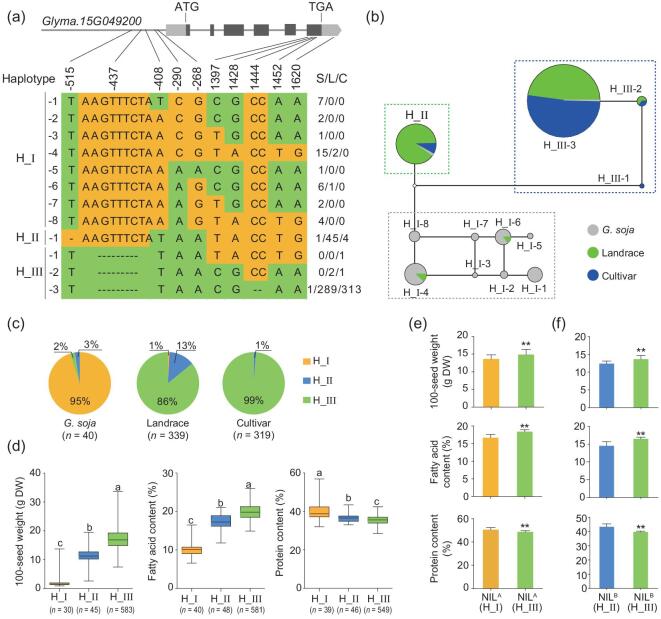
*GmSWEET10a* is a domestication gene that contributes to soybean seed size, fatty-acid content and protein content. (a) Haplotypes detected in the genomic region of *GmSWEET10a*. The SNP information of 871 re-sequenced accessions is derived from Zhou *et al*.'s data [21] and Fang *et al*.'s data [22]. The S/L/C indicates the accession number of soja/landrace/cultivar. (b) Median-joining network representing the relatedness of 12 *GmSWEET10a* haplotypes, each represented by a circle. Gray, green and blue circles represent wild soybeans, landraces and improved cultivars, respectively. (c) Frequency distribution of three haplotypes: H_I, orange; H_II, blue; H_III, green. (d) 100-seed weight, fatty-acid content and protein content of mature seeds in three haplotype populations (colors are the same as that in panel (c)). Box edges depict the interquartile range. The median is marked by a black line within the box. The number of samples in each haplotype (*n*) is shown under the haplotype label. The letters a, b and c indicate significant differences. *P* < 0.05 (Student's *t*-test). DW, dry weight. (e) and (f) Effect of two alleles of *GmSWEET10a* on seed traits. 100-seed weight, fatty-acid content and protein content of mature seeds from near-isogenic lines of *GmSWEET10a* with H_ I and H_ III haplotypes (e) or with H_II and H_ III haplotypes (f). NILs^A^ derived from the hybrid combination between HJ117 (H_I) and JY101 (H_ III). NILs^B^ derived from the hybrid combination between Suinong 14 (H_ III) and Enrei (H_II). Data are means ± s.d. ((e) NIL^A^ (H_I), *n* = 12; NIL^A^ (H_ III), *n* = 9; (f) *n* = 5). ^**^P < 0.01 (Student's *t*-test).

To verify our hypothesis, we first investigated the genetic variation of *GmSWEET10a* in wild and cultivated soybeans using our previously reported re-sequenced population [21,22]. After removing the polymorphisms with minor allele frequency <0.01 (MAF <0.01), 10 SNPs and In/Dels were found in *GmSWEET10a* in the re-sequenced population. These 10 genetic variants sorted the population into 12 haplotypes, which were represented by one to a few hundred accessions (Fig. 2a). Median-joining network analysis grouped the 12 haplotypes into three major groups, named H_I (including H_I-1 to H_I-8), H_II and H_III (including H_III-1 to H_III-3). H_I was mainly present in wild soybeans, H_II in landraces and H_III in cultivars (Fig. 2b). Allele-frequency investigation demonstrated that the proportion of H_I was significantly decreased in cultivated soybeans compared to that in wild soybeans, whereas the proportion of H_III was significantly increased in cultivated soybeans, indicating strong artificial selection of *GmSWEET10a* during soybean domestication (Fig. 2c).

Second, we looked for associations between the genetic variation of *GmSWEET10a* and seed-related traits, including seed size (indexed by 100-seed weight), protein content and oil content (indexed by total fatty acid) in the re-sequenced soybean accessions. The results showed that the seed size and the oil content of H_III were significantly higher than those of H_II and that these traits of H_II were significantly higher than those of H_I. In contrast, the protein content of H_III was significantly lower than that of H_II and that of H_II was significantly lower than that of H_I (Fig. 2d). The results indicated that selection at *GmSWEET10a* during soybean domestication pleiotropically affected the seed size, oil content and protein content. The decrease in protein content could also be a consequence of a rise in the seed size and oil content because the precursor supply may become limiting for protein synthesis when the *GmSWEET10a*-mediated sugar unloading from seed coats increases the carbohydrate state in developing embryos [32,33]. Since wild soybeans usually exhibit drastically smaller seeds, higher protein content and lower oil content than cultivated soybean (Supplementary Fig. 1), these three traits were further compared among different haplotypes only in the cultivated soybeans to eliminate the effect of genetic differences between wild and cultivated soybeans. Further, because only a few cultivated accessions had H_I haplotypes, only the differences between H_II and H_III cultivated soybeans were compared. In H_III haplotypes, the seed size and oil content were significantly higher but the protein content was lower than in H_II haplotypes (Supplementary Fig. 3).

### *GmSWEET10a* simultaneously alters the seed size, oil content and protein content

To verify whether *GmSWEET10a* simultaneously affected the seed size, oil content and protein content, two pairs of near-isogenic lines (NILs) were developed: (i) NILs^A^ carrying either H_I (NIL^A^-H_I) or H_III (NIL^A^-H_III) through a cross of HJ117 (carrying H_I) and JY101 (carrying H_III) (Fig. 2e); and (ii) NILs^B^ carrying either H_II (NIL^B^-H_II) or H_III (NIL^B^-H_III) through a cross of Enrei (carrying H_II) and Suinong 14 (carrying H_III) (Fig. 2f). Phenotypic analysis showed that NIL^A^-H_III or NIL^B^-H_III lines had significantly higher 100-seed weight and oil content and lower protein content than did NIL^A^-H_I or NIL^B^-H_II, respectively (Fig. 2e and f).

**Figure 3.**
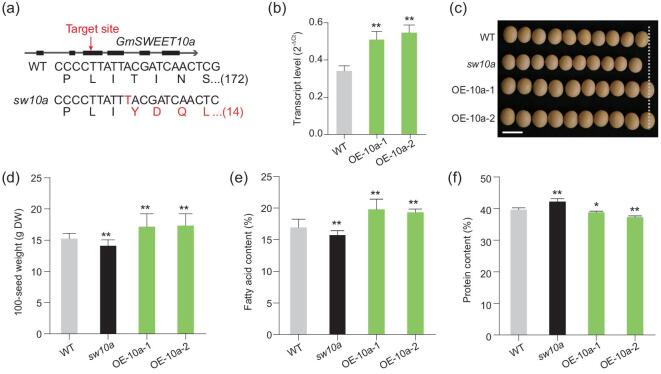
Effect of *GmSWEET10a* on seed size, fatty-acid content and protein content. (a) Genotype of the *sw10a* mutant edited by the CRISPR/Cas9 system. The arrow indicates the target site of the CRISPR/Cas9 editing in the region of exon 3 of *GmSWEET10a*. Changes in the DNA sequence in the targeted region and the amino-acid sequence of the *sw10a* mutant are highlighted in red. Numbers inside the brackets indicate the number of amino acids coded by the sequence. (b) Increased expression of *GmSWEET10a* was achieved in transgenic soybean lines OE-10a-1 and OE-10a-2 by introducing additional copies of the *GmSWEET10a* genomic sequence into the Williams 82 cultivar. (c) Seed appearance of the *sw10a* mutant, OE-10a-1 and OE-10a-2. Scale bars, 1 cm. (d)–(f) 100-seed weight (d), fatty-acid content (e) and protein content (f) of mature seeds from wild-type (WT), *sw10a* mutant, OE-10a-1 and OE-10a-2. DW, dry weight. Data are means ± s.d. ((d) *n* = 10; (e) and (f) *n* = 5). ^*^P < 0.05; ^**^P < 0.01 (Student's *t*-test).

The functions of *GmSWEET10a* were further confirmed by genetic manipulation. A knockout line, named *sw10a*, was generated by an *Agrobacterium*-delivered CRISPR/Cas9 system in the soybean cultivar Williams 82 (Fig. 3a). Compared with Williams 82, *sw10a* seeds exhibited significantly decreased seed size, lower oil content and increased protein content (Fig. 3c–f). Two independent *GmSWEET10a*-overexpression lines, OE-10a-1 and OE-10a-2, were generated by introducing an additional copy of the *GmSWEET10a* genomic sequence into the Williams 82 genome, with a significantly increased transcript level of *GmSWEET10a* (Fig. 3b). Compared with Williams 82, the seed size and oil content were significantly increased and the protein content was significantly decreased in OE-10a-1 and OE-10a-2 (Fig. 3c–f). A recent study showed that a 9-base pair deletion in the promoter of *GmSWEET10a* upregulates the expression of *GmSWEET10a*, which potentially leads to increased oil content in cultivated soybeans [34]. Here, our results in transgenic soybean clarified that the larger seed size, higher oil content and lower protein content in H_III cultivars are indeed caused by the upregulation of *GmSWEET10a*.

### Ongoing selection of *GmSWEET10b*, which is similar in function to *GmSWEET10a*

**Figure 4.**
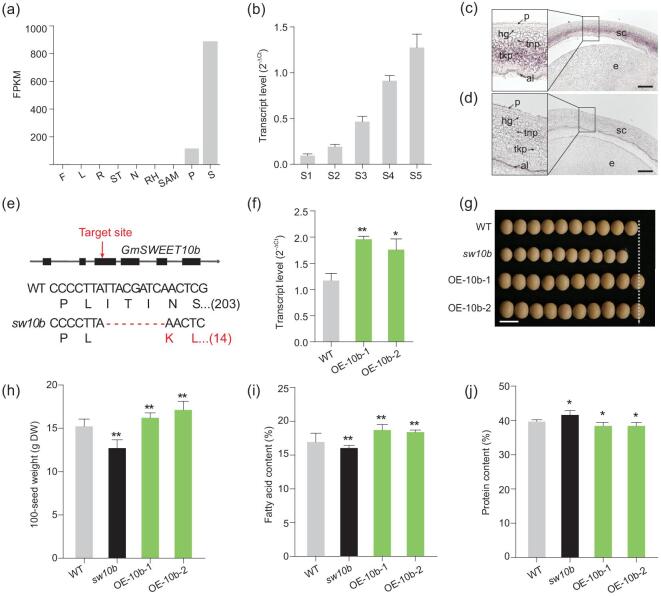
Effect of *GmSWEET10b* on seed size, fatty-acid content and protein content. (a) Expression pattern of *SWEET10b* in different organs in *Glycine max* (Gm). Expression values were obtained from Phytozome 12 (https://phytozome.jgi.doe.gov/pz/-portal.html#). F, flower; L, leaf; R, root; ST, stem; N, nodule; RH, root hair; SAM, shoot apical meristem; P, pod; S, seed; FPKM, fragments per kilobase of exon per million mapped. (b) Transcript abundance of *GmSWEET10b* in seed coats at different stages. The expression was detected by reverse transcriptase quantitative polymerase chain reaction (RT-qPCR). Transcript levels were calculated relative to soybean cyclophilin 2 (*GmCYP2*). (c) and (d) RNA *in situ* hybridization of *GmSWEET10b* showing specific expression in the seed coats. Cross-sections of developing seeds at S2–S3 hybridized with antisense (c) or sense probes (d) for *GmSWEET10b.* sc, seed coat; e, embryo; p, palisade layer; hg, hourglass; tnp, thin-walled parenchyma; tkp, thick-walled parenchyma; al, aleurone layer. Scale bars, 200 μm. (e) Genotypes of the *sw10b* mutant edited by CRISPR/Cas9 system. The arrow indicates the target site in the region of exon 3 of *GmSWEET10b*. Changes in DNA sequence in the targeted region and amino-acid sequence of the *sw10b* mutant are highlighted in red. Numbers inside brackets indicate the number of amino acids coded by the sequence. (f) Increased expression of *GmSWEET10b* was achieved in transgenic soybean lines OE-10b-1 and OE-10b-2 by introducing additional copies of the genomic sequence into the Williams 82 cultivar. (g) Seed appearance of *sw10b* mutant and overexpression lines. Scale bars, 1 cm. (h)–(j), 100-seed weight (h), fatty-acid content (i) and protein content (j) of mature seeds from wild-type (WT), *sw10b* mutant, OE-10b-1 and OE-10b-2. DW, dry weight. Data are means ± s.d. ((h) *n* = 10; (i) and (j), *n* = 5). ^*^P < 0.05; ^**^P < 0.01 (Student's *t*-test).

GmSWEET10b is a close homologue of GmSWEET10a. *GmSWEET10b* showed an expression pattern similar to, but at higher levels than, *GmSWEET10a* (Fig. 4a and b, and Supplementary Table 2). *In situ* RNA hybridization showed that *GmSWEET10b* also exhibited specific localization in the thick-walled parenchymatous layer of the seed coat, but not in the embryo (Fig. 4c and d). We investigated whether *GmSWEET10b* also played a role in controlling these three seed traits. *GmSWEET10b*-knockout and -overexpression lines were generated. The results demonstrated that *GmSWEET10b* had a similar function to that of *GmSWEET10a* (Fig. 4e–j). Moreover, a double knockout of both *GmSWEET10a* and *GmSWEET10b* in Williams 82 and Huachun 6 genetic backgrounds resulted in significantly smaller seed size, lower oil content and higher protein content than either of the single knockout lines or the WT (Williams 82) (Supplementary Fig. 4), indicating that *GmSWEET10b* and *GmSWEET10a* have functional redundancy in controlling seed development.

**Figure 5.**
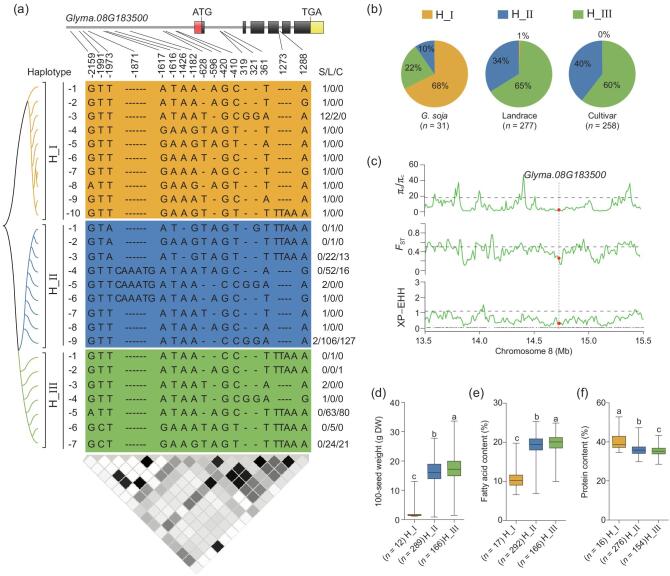
*GmSWEET10b* is a potential domestication gene that contributes to soybean seed size, fatty-acid content and protein content. (a) Haplotypes detected in the genomic region of *GmSWEET10b*. The SNP information of 871 re-sequenced accessions is derived from Zhou *et al.*'s data [21] and Fang *et al.*'s data [22]. (b) Frequency distribution of three haplotypes of *GmSWEET10b*. (c) Genetic variations (π, *F*_ST_ and XP-EHH values) were calculated between *G. soja* (S) and the cultivar (C) across the 2.0-Mb genomic region of the *GmSWEET10b* locus. The dashed horizontal lines indicate the genome-wide thresholds (top 5% of the genome) of the selection signals. The black dashed lines above the *x*-axis are annotated genes in this region. The red dots denote the *GmSWEET10b* gene—*Glyma.08G183500*. (d)–(f) 100-seed weight (d), fatty-acid content (e) and protein content (f) of mature seeds in three haplotype populations. Box edges depict the interquartile range. The median is marked by a black line within the box. The number of samples in each haplotype (*n*) is shown under the haplotype label. The letters a, b and c indicate significant differences. *P* < 0.05 (Student's *t*-test).

Similarly, the genetic variation of *GmSWEET10b* was investigated in the re-sequenced population. The nucleotide polymorphisms of this gene classified the accessions into 26 haplotypes, which were then sorted into three major groups by further phylogenetic analysis (Fig. 5a). We found that, although the ratios of H_I to H_II and H_I to H_III were greatly decreased from wild soybeans to cultivated soybeans (Fig. 5b), *GmSWEET10b* did not show significantly artificial selection during soybean domestication at the genome-wide level (Fig. 5c). However, similarly to *GmSWEET10a*, the haplotypes mainly present in cultivated soybeans exhibited significantly higher seed size and oil content but lower protein content than the haplotype mainly present in wild soybeans (Fig. 5d–f), suggesting that *GmSWEET10b* may still be undergoing selection.

### GmSWEET10a and GmSWEET10b transport sucrose and hexose, likely from seed coat to embryo

**Figure 6.**
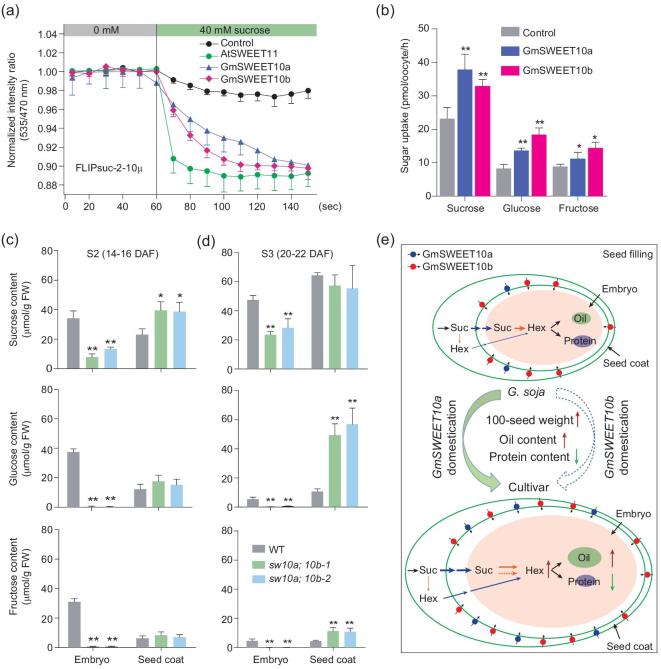
Sugar-transporter activities of GmSWEET10a and GmSWEET10b. (a) Characterization of GmSWEET10a and GmSWEET10b sucrose-transport activity using FLIPsuc-2-10μ in HEK293T. Sensor only (black) and AtSWEET11 (green) were used as negative and positive controls, respectively. Data are means ± s.d. (*n* ≥ 8). (b) Sugar-uptake transport activities of GmSWEET10a and GmSWEET10b were tested in *Xenopus* oocytes. Oocytes were injected with water (negative control), *GmSWEET10a* or *GmSWEET10b* cRNA. Data are means ± s.d. (*n* = 3). ^*^P < 0.05; ^**^P < 0.01 (Student's *t*-test). (c) and (d) Sugar content in the developing seeds at S2 (14-16 DAF) (c) and S3 (20-22 DAF) (d) stages. *sw10a;10b,* double mutants at *GmSWEET10a* and *GmSWEET10b.* Data are means ± s.d. (*n* = 3). ^*^P < 0.05; ^**^P < 0.01 (Student's *t*-test). (e) A working model for the involvement of *GmSWEET10a* and *GmSWEET10b* in seed size, oil content and protein content during soybean domestication. The expression level of *GmSWEET10a* is significantly increased in cultivars at the seed-filling stage, which promotes more hexose accumulation in the embryo, resulting in a larger seed size, higher oil content and lower protein content due to the increased carbohydrate state. Selection of *GmSWEET10b* is ongoing and might use a mechanism similar to that of *GmSWEET10a*. Dark-blue arrows indicate the translocation of sugars from the seed coat to the embryo. Orange arrows indicate the breakdown of Suc into Hex by invertase or sucrose synthase. The red and green arrows represent ‘increase’ and ‘decrease’, respectively. Hex, hexose; Suc, sucrose.

Previous studies have shown that SWEET proteins can transport either mono- or disaccharides or both [35–39]. To first test the sugar-transport activities of GmSWEET10a and GmSWEET10b, we used a newly improved, high-affinity sensor named FLIPsuc-2-10μ [40] with new N-terminal and C-terminal linkers and constructs with the 5’UTRs and codons optimized for humans. When this sensor was co-expressed with *GmSWEET10a* or *GmSWEET10b* in the human embryonic kidney line HEK293T, weak sucrose transport activity was detected (as a negative ratio change) when 40 mM sucrose was supplied (Fig. 6a). Sucrose transport by GmSWEET10a and GmSWEET10b was further confirmed by ^14^C-sucrose radiotracer uptake experiments in *Xenopus* oocytes (Fig. 6b). GmSWEET10a and GmSWEET10b can also uptake glucose and fructose in oocytes.

It is possible that the reduced seed weight in the double *sw10a;10b* mutant is caused by the low availability of sugars in embryos, similar to what is observed in *atsweet11;12;15*. To investigate this, sugar levels were measured in isolated seed coats and embryos at the end of the transition phase (14–16 DAF) and storage phase I (20–22 DAF) [41] in WT (Williams 82) and *sw10a;10b* mutants. In the embryos of the *sw10a;10b* mutants, the glucose, fructose and sucrose levels were significantly lower at both stages compared to those of WT (Fig. 6c and d). In contrast, in the seed coat of the *sw10a;10b* mutants, the sucrose content was significantly higher at 14–16 DAF and the hexose content was significantly higher at 20–22 DAF compared with those of WT. Our results indicated that the transport of these three forms of sugar from the seed coat to the embryo are impaired in the *sw10a;10b* mutant (Fig. 6c and d). This suggests that GmSWEET10a and GmSWEET10b largely determine sugar partitioning between the seed coat and the embryo.

Previous studies showed that sugar allocation affects embryo development and regulates both fatty-acid biosynthesis and protein biosynthesis [41,42]. Thus, we speculated that the increased seed size and higher oil content that arose through soybean domestication might be caused by increasing the sugar content in the embryo through the selection of elite *GmSWEET10a* alleles.

## DISCUSSION

Seed size, oil content and protein content are essential factors for soybean yield and quality. Each of these quantitative traits is controlled by multiple genetic loci. At least 267, 299 and 225 QTLs have been reported to be responsible for the seed size, oil content and protein content in soybean, respectively (retrieved from SoyBase, https://soybase.org/). In the study, we have shown that GmSWEET10a and GmSWEET10b are specifically expressed in the seed coat, likely transport sugars from seed coats to embryos and genetically regulate seed size and composition. *GmSWEET10a* is a QTL that genetically regulates seed size and composition simultaneously, and was subjected to strong artificial selection during soybean domestication. However, plots of the 100-seed weight against the fatty-acid content and the protein content from the natural population showed that there are cultivated soybeans combining the traits of large seed and high oil content or the traits of large seeds and high protein content (Supplementary Fig. 1d and e). Thus, selections on other genes that do not have pleiotropic effects on these three traits likely occur.

A working model of *GmSWEET10a* and *GmSWEET10b* function and their contribution to soybean domestication is proposed (Fig. 6e). At the seed-storage stage, sucrose, as the major carbon source, is delivered to the seed coat via the funicular phloem. Then sucrose, together with a few hexoses (including glucose and fructose) presumably hydrolysed from sucrose, is exported into the cell-wall space via GmSWEET10a and GmSWEET10b, and subsequently imported into the embryo by other sugar transporters [43]. Imported sugars are metabolized for energy generation and carbon skeleton supply for the synthesis of storage compounds including lipids, proteins and starch.

Sucrose concentrations in seed coats and embryos reach a high and steady level at the rapid-seed-growth stage [44]. Thus, sucrose flux across seed coats is particularly important to meet the increasing demand for a carbon source for a high rate of seed growth. Haplotype III of *GmSWEET10a* was selected during soybean domestication (from *G. soja* to *G. max*) because it confers a relatively higher expression of *GmSWEET10a*, which allows more sucrose to flux to the developing embryos at the rapid-seed-growth stage, and consequently leads to a higher seed-growth rate, larger seed and higher oil content. The selection of *GmSWEET10b* is currently ongoing and presumably leads to a selected function in contributing to seed-size-storage components, like *GmSWEET10a*.

The positive contribution of *GmSWEET10a* and *10b* to seed size and oil content can be attributed to the following reasons. First, the elevated expression of the sugar transporter *GmSWEET10a* or *GmSWEET10b* can lead to the flux of more sugars into embryos from maternal tissues. It may trigger embryo cell division and expansion, and consequently larger seeds. Second, the increased transport of sugars into the embryos would result in an increase in the carbon resources for lipid synthesis. Some intermediates derived from glycolysis are directly or indirectly shared by lipid- and protein-synthesis pathways. Lipid synthesis may be enhanced due to more precursor of acetyl-CoA available from glycolysis and thus more lipid can be produced and accumulate. On the other hand, protein synthesis depends on both carbon and nitrogen availability. As GmSWEET10a or GmSWEET10b sugar-transporter activities increase, the nitrogen availability may become a limiting factor and thereby decrease the relative protein contents.

The knocking out of *GmSWEET10a* resulted in a 7.4% and 7.2% decrease in the 100-seed weight and fatty-acid content, but a 6.4% increase in the protein content (Fig. 3d–f). A similar effect was observed for its homologue *GmSWEET10b* (Fig. 4h–j). When both *GmSWEET10a* and *GmSWEET10b* were knocked out, the impact on these parameters increased to –40.2%, –40.7% and +32.1%, respectively (Supplemental Fig. 4b–d and f–h). These seed phenotypes supported that GmSWEET10a and GmSWEET10b are essential sugar transporters for sugar unloading from the soybean-seed coat to the embryos. It is worth noting that the knockout of *GmSWEET10b* has a stronger impact on the seed weight, as well as the oil and protein content, compared with *GmSWEET10a* (Figs 3d–f and 4h–j). This indeed is consistent with the transcript-level difference in their transcript abundance (Figs 1c and 4b), although not proportionate to the 10-fold difference at their transcript levels. It requires further study to determine whether their protein abundance and transporter activities correspond to their transcript abundance.

In addition to that, we retrieved the expression data of all the members of the *SWEET* and *SUT/SUF* family, which includes genes that have been implicated in exporting sucrose from the seed coat in pea and bean [45,46] in the seed coats from Gene Networks in Seed Development (Supplemental Table 2). Among the 27 *SWEET* and *SUT/SUF* genes analysed, only *GmSWEET10s*, *GmSWEET13s* and *GmSWEET14s* were expressed in seed coats. RT-qPCR showed that *GmSWEET13s* and *GmSWEET14s* were indeed expressed in seed coats, although at a lower level than in *GmSWEET10a* and *GmSWEET10b* (Supplemental Fig. 5a and b). Thus, whereas we speculate that, while GmSWEET10a and GmSWEET10b play essential roles in sugar unloading from the seed coats to the embryos, other uncharacterized sugar transporters, such as GmSWEET13s and GmSWEET14s, may also play roles, either due to their inherent function or as a compensation mechanism in the absence of *GmSWEET10a* and *GmSWEET10b*.

Introduction of an additional genomic copy of either *GmSWEET10a* or *GmSWEET10b* into soybean led to significant increases in yield, ranging from 11% to 20%, without the compromise of other agronomic traits (Supplementary Fig. 6). Genome editing is a powerful approach for targeted mutagenesis and has been successfully used for crop-trait improvement [47–50]. A recent study found that disruption of *MIR396e* and *MIR396f* by CRISPR/Cas9 significantly improves rice yield under nitrogen-deficient conditions [51]. We speculate that alteration of the expression of *GmSWEET10a* and *GmSWEET10b* by precise genome editing [52] may enhance seed and oil yield in soybean. Furthermore, since *GmSWEET10b* has not yet been fixed in cultivated soybeans, the further discovery and utilization of elite allele(s) of *GmSWEET10b* may provide a new avenue for future soybean breeding. Another member of the *SWEET* family, *SWEET4*, was found to be likely selected during the domestication of both maize and rice [15], indicating that a parallel selection of the *SWEET* family members may exist across different crop species during domestication. Identification of these genes would facilitate the improvement of current crops [53,54]. Thus, *SWEET* genes should be priority targets across a wide range of species for the improvement of crops and possibly even underused or undomesticated plants.

## MATERIALS AND METHODS

For details, please see Supplementary data.

## Supplementary Material

nwaa110_Supplemental_File
