# Supplementary material for: Simultaneous changes in seed size, oil content and protein content driven by selection of SWEET homologues during soybean domestication
Source: Natl Sci Rev. 2020 May 27;7(11):1776–86. doi: 10.1093/nsr/nwaa110 (PMC8290959; doi:10.1093/nsr/nwaa110)
Supplement: nwaa110_Supplemental_File [file nwaa110_supplemental_file.zip › nwaa110-Supplemental_Figures_and_Tables.pdf]

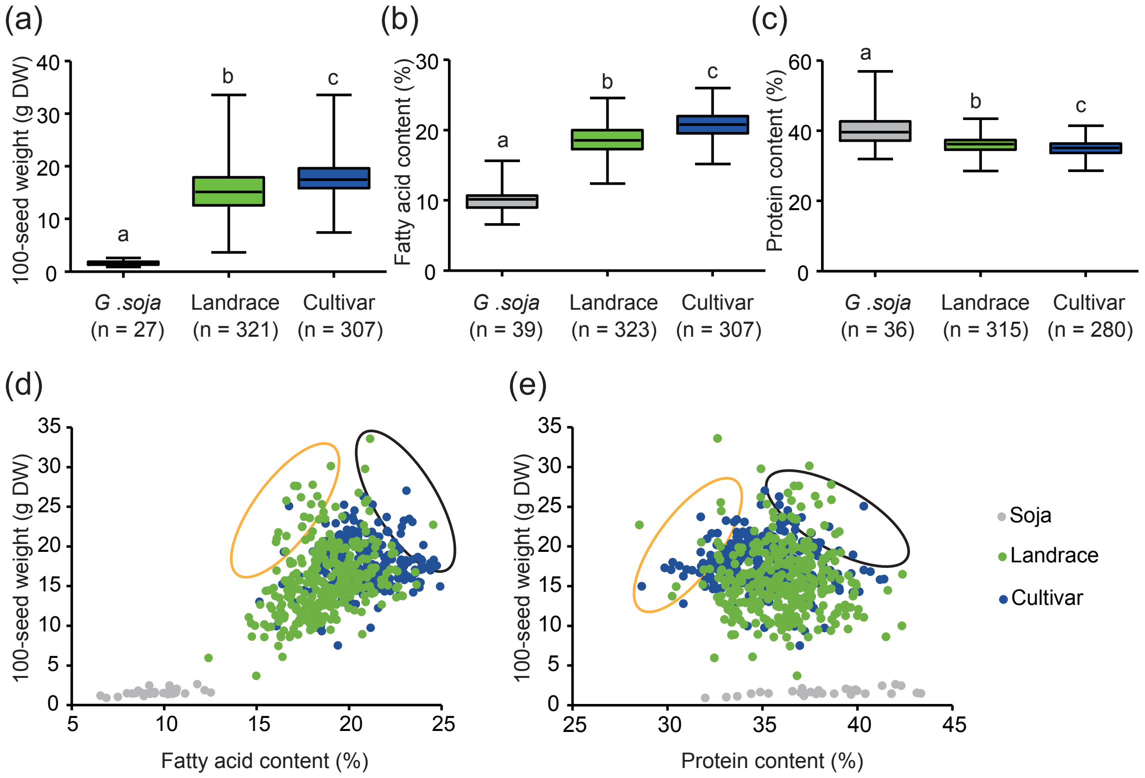

Supplementary Figure 1. Comparison of seed-related traits in different subgroups of soybean germplasm. (a-c) 100-seed weight (a), fatty acid content (b), and protein content (c) of mature seeds in *G. soja*, landraces, and cultivars. (d) 100-seed weight plots against fatty acid content. (e) 100-seed weight plots against protein content. DW, dry weight. Box edges depict interquartile range. The median is marked by a black line within the box. Number of samples in each haplotype (n) is shown under the haplotype label. The letters a, b and c indicate significant differences. *P* <0.05 (Student's *t*-test).

(a)

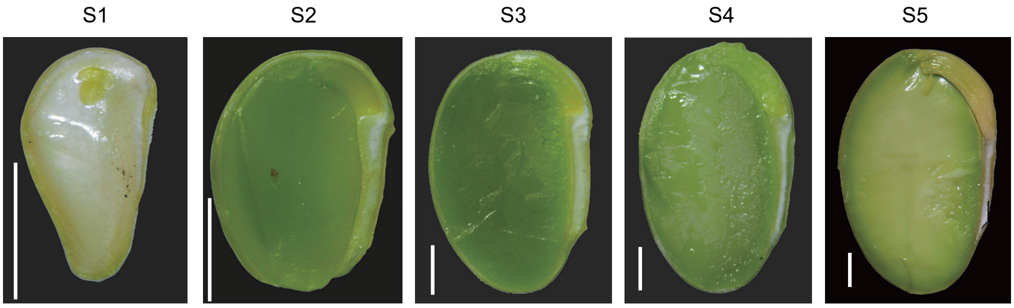

(b)

Sampling time		Developmental stage and events	Fresh weight
S1	Six to 7 days post flowering	Heart stage: Cotyledons begin development and are just visible	NA
S2	Fourteen to 16 days post flowering	Seed filling stage 1: Endosperm is completely assimilated, embryo occupies whole cavity of seed coat. Seed filling is just beginning.	10-20 mg
S3	Twenty to 22 days post flowering	Seed filling stage 2: Green seeds with 90-100mg fresh weight (around 1/3 of final size); accumulation in nutrients, oil, storage protein in cotyledon	90-100 mg
S4	Twenty-seven to 30 days post flowering	Seed filling stage 3: Green seeds with 140-160 mg fresh weight (around 1/2 of final size)	140-160 mg
S5	Fourty-two to 48 days post flowering	Full seed stage: Green seeds that fills the pod cavity	350-380 mg

Supplementary Figure 2. Sampling timepoints. (a) Longitudinal sections of the development seeds at different stages. Scale bars, 1 mm. (b) Description of sampling timepoints.

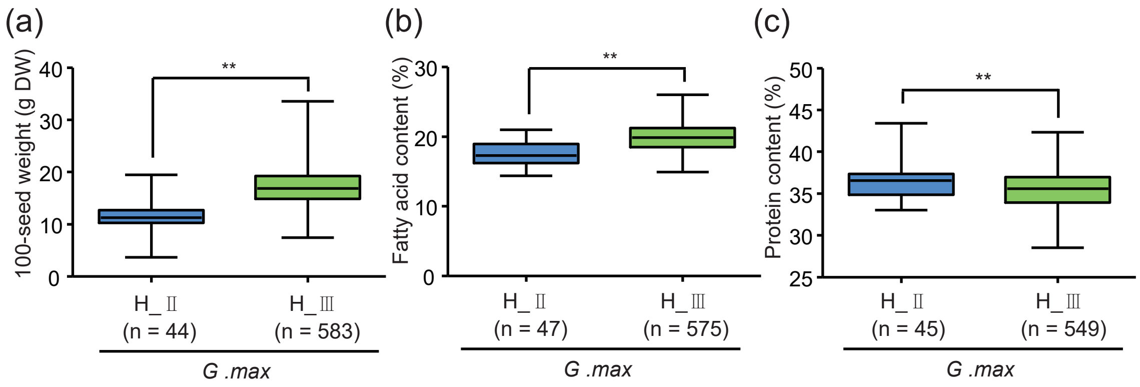

Supplementary Figure 3. Comparison of seed-related traits in two haplotype populations of cultivated soybean (*G. max*). (a-c) 100-seed weight (a), fatty acid content (b), and protein content (c) of mature seeds in two haplotype populations. DW, dry weight. Box edges depict interquartile range. The median is marked by a black line within the box. Number of samples in each haplotype (n) is shown under the haplotype label. **$P < 0.01$ (Student's *t*-test).

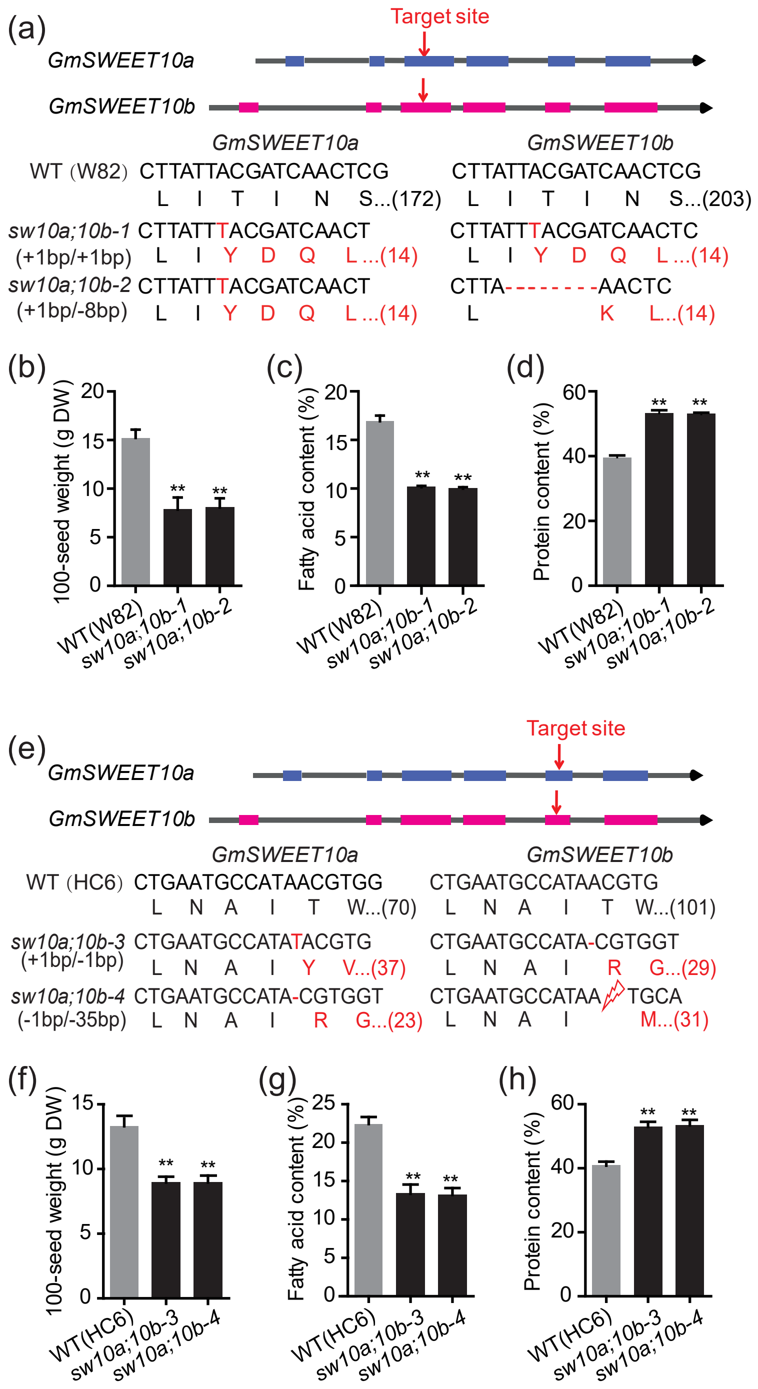

Supplementary Figure 4. Phenotypes of *sw10a;10b* mutants. (a) Genotypes of the *sw10a;10b* mutants generated by CRISPR/Cas9 system in the Williams 82 background. The red arrows indicate the target site in the conserved region of the 3rd exon of *GmSWEET10a* and *GmSWEET10b*. Changes in the DNA sequence in the targeted region and amino acid sequence of the *sw10a;10b* mutants are highlighted in red. Numbers inside the brackets indicate the number of amino acids coded by the sequence. (b-c) 100-seed weight (b), fatty acid content (c) and protein content (d) of mature seeds from wild type (W82) and *sw10a;10b* mutants grown in the field. (e) Genotypes of the *sw10a;10b* mutants generated by CRISPR/Cas9 system in Huachun 6 background. The red arrows indicate the target site in the conserved region of the 5th exon of *GmSWEET10a* and *GmSWEET10b*. Changes in the DNA sequence in the targeted region and amino acid sequence of the *sw10a;10b* mutants are highlighted in red. (f-h) 100-seed weight (f), fatty acid content (g) and protein content (h) of mature seeds from wild type (HC6) and *sw10a;10b* mutants grown in field. DW, dry weight. W82, Williams 82. HC6, Huachun 6. Data are means ± s.d. (b, n = 10; c and d, n = 5; f-h, n = 10). $^{**}P < 0.01$ (Student's *t*-test).

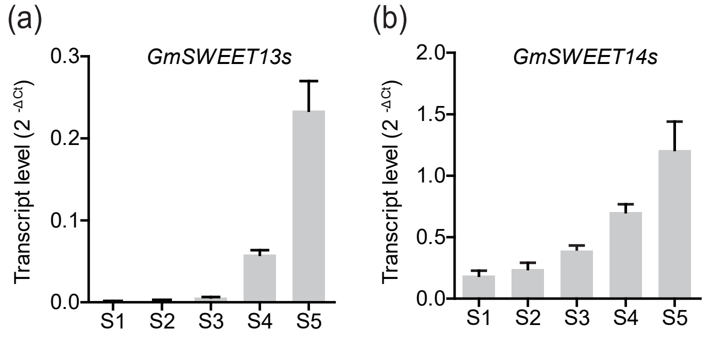

Supplementary Figure 5. Transcript abundance of *GmSWEET13a/b/c/d* and *-14a/b* in seed coats at different stages. The expression was detected by reverse transcriptase quantitative polymerase chain reaction (RT-qPCR). Transcript levels were calculated relative to soybean cyclophilin 2 (*GmCYP2*). DAF, days after fertilization. Data are means ± s.d.

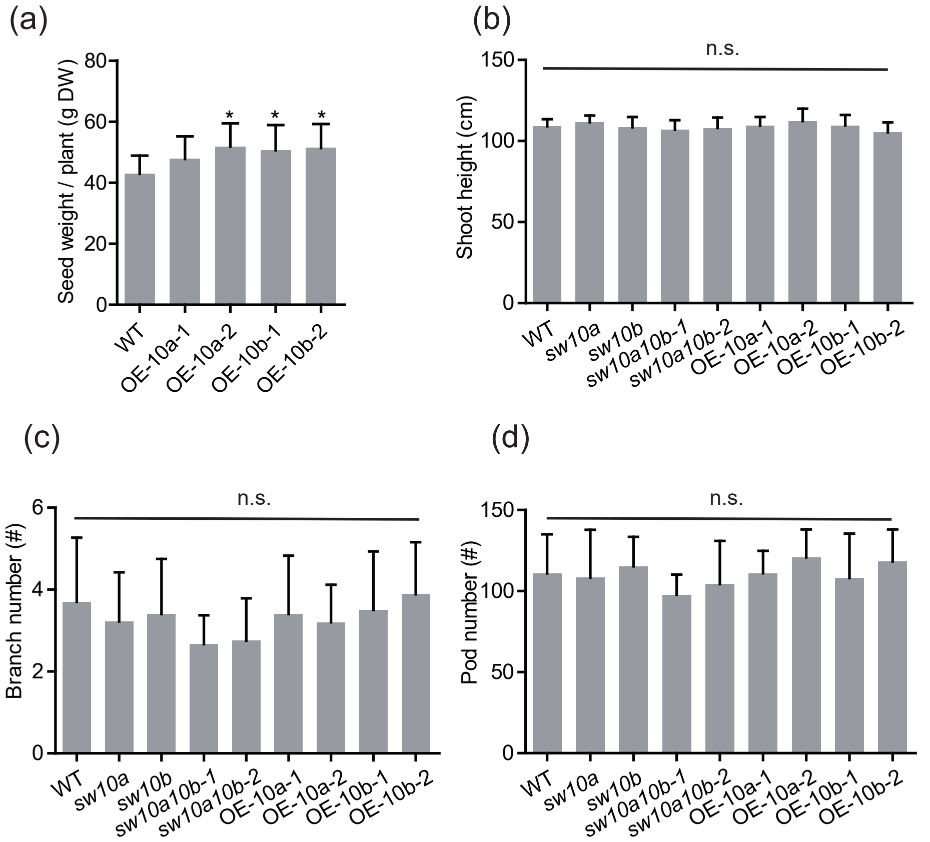

Supplementary Figure 6. Phenotypes of plant architecture-related traits. (a) Seed weight per plant in *GmSWET10a* and *GmSWET10b* overexpression lines grown in field. Data are means ± s.d. (n = 9). *$P < 0.05$ (Student's *t*-test). (b-d) Shoot height (b), branch (c) and pod (d) number of WT and all transgenic plants used in this study grown in the field. Data are means ± s.d. (n = 10). n.s., not significant (Student's *t*-test).

Supplementary Table 1. QTLs information.

QTL name	Chromosome 15 (Mb)	Trait name	Reference
Seed volume 1-1	1.30~4.41	Seed size	25
q100SW15	3.72~4.05	Seed size	28
Seed length 1-1	1.30~4.41	Seed width	25
Seed protein 30-3	3.31~6.84	Protein content	24
qPro15	3.72~4.05	Protein content	28
cqSeed oil-007/010	3.31~3.99	Oil content	27
qOil15	3.72~4.05	Oil content	28
Seed oil 32-1	3.44~6.84	Oil content	26

Supplementary Table 2. Expression profiles of sugar metabolism related genes in early development seeds. The data was extracted from Gene Networks in Seed Development (http://seedgenenetwork.net/soybean) (RPKM > 1). SUT, sucrose transporters; SUF, sucrose facilitator.

	Gene name	Axis						Cotyledon				Seed Coat			
		Shoot Meristem	Plumule	Parenchyma	Root Meristem	Vascular Bundle	Epidermis	Adaxial Parenchyma	Vascular Bundle	Adaxial Epidermis	Aleurone	Parenchyma	Hourglass	Palisade	Hilum
GmSWEET	GmSWEET10b	20.7	0.8	0.6	3.2	0.7	4.4	8.7	7.4	54.7	135.9	10895.3	139.1	7.1	70.8
	GmSWEET10a	0.4	0.0	0.0	0.0	0.1	0.1	1.1	0.1	9.9	23.6	1362.5	4.5	0.8	0.1
	GmSWEET14a	0.0	0.0	0.0	0.0	0.1	0.0	0.1	0.1	0.3	0.2	1235.8	6.7	0.4	0.0
	GmSWEET14b	0.1	0.1	0.0	0.0	0.0	0.0	0.0	0.1	0.5	0.3	1134.3	7.7	0.9	0.0
	GmSWEET13a	0.0	0.0	0.0	0.0	0.0	0.0	0.0	0.0	0.1	0.2	297.6	0.9	0.0	0.2
	GmSWEET13b	0.0	0.0	0.0	0.0	0.0	0.0	0.1	0.1	0.0	0.1	261.5	2.4	0.0	0.1
	GmSWEET13c	0.0	0.0	0.0	0.0	0.0	0.0	0.0	0.0	0.0	0.0	44.4	0.6	0.0	0.0
	GmSWEET13d	0.0	0.0	0.0	0.0	0.0	0.0	0.0	0.0	0.0	0.0	33.8	0.2	0.0	0.0
	GmSWEET7	68.9	11.3	56.3	36.5	13.1	220.9	40.5	10.9	284.4	196.0	3.2	0.2	0.5	0.0
	GmSWEET11	0.0	0.0	0.0	0.0	0.0	0.0	0.0	0.0	0.1	0.1	0.0	3.3	0.1	290.6
	GmSWEET1c	0.0	3.8	5.6	0.0	0.0	0.4	1.4	0.0	0.1	0.0	0.2	59.8	0.6	0.3
	GmSWEET2a	2.6	1.4	1.1	3.1	0.7	1.5	1.0	0.6	1.4	1.0	1.6	0.2	1.5	0.8
	GmSWEET2b	3.2	1.2	2.5	2.2	1.9	1.3	1.7	2.6	1.4	1.1	1.0	1.4	2.3	3.1
	GmSWEET2c	3.4	1.3	2.6	1.0	4.7	2.0	2.5	11.4	5.9	2.5	7.2	5.1	2.3	3.0
	GmSWEET3a	0.0	0.0	0.0	0.0	0.0	0.0	0.0	0.0	0.0	0.0	2.1	0.0	0.0	0.0
	GmSWEET3b	0.0	0.2	0.0	0.0	1.2	0.0	0.0	9.6	0.0	0.0	3.6	0.0	0.0	0.0
	GmSWEET6	0.2	0.6	0.0	0.2	0.1	0.3	0.1	0.0	2.0	0.3	7.0	71.1	0.6	0.0
	GmSWEET17	0.5	0.2	0.1	0.1	0.6	0.3	0.8	1.5	1.6	0.4	1.1	2.8	3.3	1.3
	GmSWEET15a	0.1	0.0	0.1	0.7	0.0	0.0	0.1	0.2	2.6	332.3	0.6	0.0	0.1	0.0
	GmSWEET15b	0.1	0.0	0.2	0.1	0.0	0.1	0.0	0.0	2.0	215.6	0.2	0.1	0.0	0.0
GmSUT / SUF	Glyma02g08250	1.5	0.8	0.2	0.4	0.0	5.9	4.3	1.1	3.6	63.4	20.9	60.3	8.1	1.9
	Glyma02g08260	2.8	1.6	5.9	0.2	0.1	6.4	10.7	2.3	9.6	0.4	1.1	0.1	0.0	0.0
	Glyma02g38300	2.6	2.7	2.3	2.9	3.1	1.7	1.6	4.1	1.8	4.2	7.8	2.8	0.8	4.6
	Glyma04g09460	1.6	1.7	1.9	2.5	2.2	3.2	3.3	3.9	3.3	3.1	1.2	1.9	1.2	2.0
	Glyma08g40980	8.6	3.0	3.8	6.2	4.1	6.1	5.5	8.5	5.3	5.6	5.2	9.9	7.4	12.3
	Glyma10g36200	3.9	0.5	1.5	11.0	0.0	60.1	0.2	0.7	171.1	16.4	0.2	0.4	0.2	0.2
	Glyma16g27320	0.0	0.0	0.0	0.1	0.0	0.1	0.1	0.0	0.3	5.7	0.1	0.0	0.0	0.0

Supplementary Table 3. Primers used in this study.

Primer name	Sequence (5' to 3')	Primer use
**For genotype identification**		
10a-F	TTGATTTTGAAATTCAAATCC	Genotyping for *GmSWEET10a* with different haplotypes
10a-R	ACTTGGCAATTAATCCTTGGC	
CAS9-10a-F	CATTTGCAATTCTCGGGTCAT	Genotyping for *GmSWEET10a* in the mutant (W82 background)
CAS9-10a-R	TAAGGAATGGAATTTTTGACTC	
CAS9-10b-F	TTTCTTAACAGGCGAATGCT	Genotyping for *GmSWEET10b* in the mutant (W82 background)
CAS9-10b-R	CTTGCATCCTTGTGCGCATA	
GmSWEET10a-F	GTGTCACTAGCAAGCTAACTCTC	Genotyping for *GmSWEET10a* in the mutant (HC6 background)
GmSWEET10a-R	CCTTCTCACTCTCACCGCCG	
GmSWEET10b-F	TGGATTTGTGACGCCGTTTCA	Genotyping for *GmSWEET10b* in the mutant (HC6 background)
GmSWEET10b-R	CCTTCTCACTCTCACTGCCC	
**For RT-qPCR**		
10a-QRT-F1	GCAAGCTTTAGCTGAAGGAGCGAT	RT-qPCR for *GmSWEET10a*
10a-QRT-R1	TCATCCACTTCCTCTGCGATTGAA	
10b-QRT-F	CCTGCTGAAGTCTTCCCAAT	RT-qPCR for *GmSWEET10b*
10b-QRT-R	GGCAATCATCCTTGGCTTCC	
13a/b/c/d-QRT-F	GGTTCTTCTATGGCCTTCTC	RT-qPCR for *GmSWEET13a/b/c/d*
13a/b/c/d-QRT-R	ATAAACCAAATACAGCACCATC	
14a/b-QRT-F	GCTGTTATGTGGTTCTTCTATG	RT-qPCR for *GmSWEET14a/b*
14a/b-QRT-R	GCGTTTCTGTACATCAAATACA	
CYP2-QRT-F	CGGGACCAGTGTGCTTCTTCA	RT-qPCR for GmCYP2
CYP2-QRT-R	CCCCTCCACTACAAAGGCTCG	
**For in situ hybridization**		
antisense *GmSWEET10a*-F	CATCCCCCATTCATTCACAAG	To amplify the antisense probe of *GmSWEET10a*
antisense *GmSWEET10a*-R	GTAATACGACTCACTATAGGGC-TATTCATGGCCGCGAATAGC	
antisense *GmSWEET10b*-F	AAGGGCAGTGAGAGTGAGAAG	To amplify the antisense probe of *GmSWEET10b*
antisense *GmSWEET10b*-R	GTAATACGACTCACTATAGGGC-GAGGAGCAGAATGAAGTAAAG	
sense*GmSWEET10a*-F	GTAATACGACTCACTATAGGGC-CATCCCCCATTCATTCACAAG	To amplify the sense probe of *GmSWEET10a*
sense*GmSWEET10a*-R	TATTCATGGCCGCGAATAGC	
sense*GmSWEET10b*-F	GTAATACGACTCACTATAGGGC-AAGGGCAGTGAGAGTGAGAAG	To amplify the sense probe of *GmSWEET10b*
sense*GmSWEET10b*-R	GAGGAGCAGAATGAAGTAAAG	
**For vector construction of overexpression**		
p10a-g10a-F	gtcgactctagagctagagTAAGCGTCAAGACAGGTTT	p*GmSWEET10a*-g*GmSWEET10a* vector
p10a-g10a-R	cggggaaattcgagctcgTCATCCACTTCCTCTGCGATTG	
p10b-g10b-F	gcatgcctgcaggtcgactTGCACATAACACAAATAGCA	p*GmSWEET10b*-g*GmSWEET10b* vector
p10b-g10b-R	cggggaaattcgagctcgTCACACTGGGCAATCATCCTT	
