# Supplementary material for: Simultaneous changes in seed size, oil content and protein content driven by selection of SWEET homologues during soybean domestication
Source: Natl Sci Rev. 2020 May 27;7(11):1776–86. doi: 10.1093/nsr/nwaa110 (PMC8290959; doi:10.1093/nsr/nwaa110)
Supplement: nwaa110_Supplemental_File [file nwaa110_supplemental_file.zip › nwaa110_Supplementary_data_Methods.docx]

**MATERIALS AND METHODS**

**Plant materials and growth conditions**

Cultivated soybean (*Glycine. max* [L.] Merr.) and wild soybean (*G. soja* Sieb. & Zucc.) were used in this study. Cultivar Williams 82 and Huachun 6 seeds were used as the recipients for *Agrobacterium tumefaciens*-mediated transformation. Other than the field experiments indicated below, soybean plants were grown in pots with nutritional soil (Pindstrup, Denmark) in greenhouse (16-h-light/8-h-dark, 30°C day/25°C night).

The pair of near-isogenic line NILs^A^, NIL^A^ (H_I) and NIL^A^ (H_III), were developed from a cross of JIHJ117 (HJ117, carrying H_I) and Jiyu101 (JY101, carrying H_III). A heterozygous line at the *GmSWEET10a* were selected in the progenies at F7 generation and the two individual segregating line with different homozygous alleles of NIL^A^ (H_I) and NIL^A^ (H_III) at F8 generation were used as NIL pair. The NILs^B^ lines, NIL^B^ (H_II) and NIL^B^ (H_III), were developed using similar method from a cross of Enrei (carrying H_II) and Suinong 14 (carrying H_III).

For field experiments, transgenic plants with Williams 82 background, NILs^B^ and soybean accessions with different haplotypes were grown in every June in Anhui Academy of Agricultural Science (Hefei, Anhui, China) (E117.25, N31.89). Transgenic plants with Huachun 6 background were planted in the university farm in Fuzhou, Fujian, China (E119.24, N26.08) in 2019. NILs^A^ were planted in the field in Shijiazhuang, Hebei, China (E114.78, N37.91).

**Plasmid construction**

**Generation of CRISPR/Cas9 mutations**

CRISPR/Cas9 mutations of *GmSWEET10a* and *GmSWEET10b* in Williams 82 was conducted at Zhejiang University followed the procedure previously described [20]. The target was designed using the online tools CRISPRP v2.0 (http://crispr.hzau.edu.cn/cgi-bin/CRISPR2/CRISPR) based on their GC content and putative off target sites. The 20-bp target sequence (5’-GATCGAGTTGATCGTAATAA-3’) was designed in the conserved region of both *GmSWEET10a* and *GmSWEET10b* in the third exon. The target sequence was synthesized and cloned into pBlu gRNA (containing U6 promoter) at *Bbs*I site, the generating construct was then digested with *EcoR*I, the fragment containing gRNA was transferred to Cas9 MDC123 at same enzyme site (Cas9-*GmSWEET10*). CRISPR/Cas9 mutations in Huachun 6 was generated at Fujian Agriculture and Forest University using the protocol published [54]. For sgRNA design, the 20-bp target sequence (5’-TCACACTGAATGCCATAACG-3’) was designed in the conserved region of both *GmSWEET10a* and *GmSWEET10b* in the fifth exon.

For determining the mutations in the target gene, fragments flanking the target site were amplified by PCR using the primers **(Supplementary Table 3)** and the PCR products were purified for Sanger sequencing to detect potential mutations.

**Overexpression of *GmSWEET10a* and *GmSWEET10b***

The endogenous promoters were used to overexpress *GmSWEET10a and GmSWEET10b* in situ in soybean. For overexpression of *GmSWEET10a*, 3453-bp fragment of genomic sequence containing a 1989-bp promoter (upstream ATG of *GmSWEET10a*) and 1464-bp entire coding region sequence (including the intron) was cloned into modified pBI121 vector (p*GmSWEET10a*-g*GmSWEET10a*). For overexpression of *GmSWEET10b*, 3894-bp fragment of genomic sequence containing a 2086-bp promoter (upstream ATG of *GmSWEET10b*) and 1808-bp entire coding region sequence (including the intron) was cloned into modified pBI121 vector (p*GmSWEET10b*-g*GmSWEET10b*).

The above constructs were introduced into *Agrobacterium* strain LBA4404 or GV3101 for soybean transformation*.*

**In situ hybridization**

For *in situ* hybridization, the digoxigenin-labeled antisense or sense probes were transcribed and labeled using Roche DIG RNA Labeling kit (Roche, Mannheim, Germany). The primers to amplify the sense and antisense probes of *GmSWEET10a* and *GmSWEET10b* were listed in Supplementary Table 3. Developing seeds at S2-S3 were collected and fixed in FAA [formaldehyde: glacial acetic acid: ethanol, 3.7:5:50% (v/v/v)] for 48 h at 4°C. Samples dehydration, embedment, sectioning and *in situ* RNA hybridization was performed as described previously [55].

**Genetic diversity analysis**

SNP data from our previous study [21] were used for the genetic diversity analysis of *GmSWEET10a* and *GmSWEET10b* in soybean. The SNPs with missing data > 10% or MAF < 5% were filtered. The soybean accessions were divided into three populations: *G. soja*, landrace, and cultivar. π was calculated using a 20-k-2-k sliding window. After filtering the windows with < 10 SNPs in wild and 0 SNPs in cultivated populations, we calculated the ratio of diversity (π_wild_/π_cultivated_) for each window. *F*_ST_ values were calculated with a 20-k-2-k sliding window using VCF tools [56] to calculate the pairwise genomic differentiation for wild and cultivated populations of soybean. The extended haplotype homozygosity test (XP-EHH) was implemented by R pack REHH2.0 [57] using a 20-k-2-k sliding window in both the wild and cultivated populations. The top 5% genome sequences were determined as selective sweeps.

**RNA extraction and reverse transcription quantitative PCR (RT-qPCR)**

To analyze the expression pattern of *GmSWEET10a* and *GmSWEET10b*, soybean plants were grown in pots in greenhouse. Seed coats from developing seeds at S1 to S5 stage were collected and ground in liquid nitrogen by a tissue homogenizer (TL2010S, DHS, China). Total RNA extraction and cDNA synthesis were performed as previously described [20]. Primers used for *GmSWEET10a* and *GmSWEET10b* were specific to each of the genes **(Supplementary Table 3)**. RT-qPCR was conducted using SYBR premix Ex Taq (RR420, Takara, Japan) with a LightCycler^®^ 480 machine (Roche, Germany). Soybean cyclophilin *CYP2* was used as an internal control [58]. Transcript levels were calculated relative to *GmCYP2* using the equation 2^-ΔCt^. The primers used for RT-qPCR are shown in Supplementary Table 3. All amplification reactions were performed with three or four biological repetitions and two technical replicates.

**Soluble sugar analyses**

For determination of soluble sugar content, developing seeds of *sw10a;10b* mutants and WT grown in greenhouse were collected at 14-16 (S2 stage) or 20-22 DAF (S3 stage). Fifty to seventy milligrams of separated embryos and seed coats were sampled and frozen into the liquid nitrogen. Extraction of sucrose, glucose and fructose was carried out according to the previous reported method [20]. The sugar composition was analyzed using an ion chromatography system (ICS-3000, Dionex, USA). The machine was equipped with a 4 × 250 mm column (CarboPac PA1, Dionex, USA), and the column temperature was controlled at 30°C. Sodium hydroxide solution (200 mM) was used as eluent with flow rate at 1 ml min^-1^. Twenty-five microliter sample was injected using an AS-DV auto-sampler (Dionex, USA) and detected using an ED 40 electrochemical detector with Ag/AgCl reference electrode and carbohydrates waveform (quadruple potential). According to the retention times, the peaks in order are as follows: glucose, fructose and sucrose. The concentration of each soluble sugar was analyzed by the standard curve.

**Seed fatty acid analysis**

For total fatty acid determination, a quantitative method was performed through heated-methylation extraction method and gas chromatography analysis as previous described with minor changes [59]. Dried mature seeds were ground into powder and transferred into 15 ml glass tube followed with 2 ml extraction buffer (chloroform: isopropanol, 2:1). Fatty acid methyl ester was generated after treated with 2 ml 1% sulfate in methanol (v/v) at 80°C for 1 h. Then FAME was extracted using 3 ml hexane and 1 mL 0.9% (w/v) NaCl. One mL hexane phase was used for FAME detection on a gas chromatography equipped with a DB-23 column (Agilent, CA, USA). The heating program including three procedures: 1, 120°C for 5 min; 2, increased by 4°C min^-1^ to 190°C and held for 12 min; 3, continued to increase to 210°C at 2.5°C min^-1^ and maintained for 10 min at the final temperature. One microliter sample was injected and detected by a gas chromatography-flame ionization detector (7890A, Agilent, USA) at 280°C.

**Seed protein content analysis**

Seed protein content was determined by the modified Kjeldahl method [60]. Sample with 200 mg heat-dried seed powder was digested by 15 ml H_2_SO_4_ and composite catalyst (CuSO_4_ and K_2_SO_4_) overnight. Then the samples were treated with four steps: 160°C, 15 min; 220°C, 30 min; 350°C, 30 min; 450°C, 120 min. Nitrogen content was analyzed using an automatic Kjeldahl apparatus (Kjeltec^TM^ 8400, FOSS, Denmark). Percentage protein was calculated by percentage N multiplied by the factor 6.25.

**Constructs and imaging in HEK293T cells**

To maximize gene expression in mammalian cell lines, the codons of *GmSWEET10a* and *GmSWEET10b* ORF including 5’UTR were optimized based on codon preference in human cell lines and synthesized (Genscript, Nanjing, China). They were cloned into pDORN201 and then transferred into the mammalian expression vector pcDNA3.2/V5-DEST (Invitrogen, CA, USA) using the Gateway cloning strategy. To improve the dynamic range of the high affinity sucrose sensor FLIPsuc-10µ [35], the N-terminal linker (DVGMDEGTGGA) before the binding protein and the C-terminal linker (GAGDV) after the binding protein were used. The sensor was cloned into pcDNA3.2/V5-DEST too. The transport activity was analyzed as described before [37]. Briefly, HEK293T cells were transfected as described before with minor changes in transfection reagent. Briefly, HEK293T cells were co-transfected with the plasmid carrying the sensor FLIPsuc-2-10μ and a plasmid carrying GmSWEET10a, GmSWEET10b or AtSWEET11(a positive control) using TurboFect^TM^ Transfection Reagent (Thermo Scientific^TM^) in 96-well plates Imaging process followed the procedure described in Wang paper [61]. Two days after transfection and 30min before imaging, cells were washed with Hanks Balanced Saline Salt buffer and kept in Hanks buffer. 60 μL of 80 mM sucrose was added to the well to reach a final sucrose concentration of 40 mM after 2 min of imaging. Imaging was conducted using a Zeiss Axio Oberve Z1/7 with Hamamatsu camera ORCA-Flash4.0 V3 Digital CMOS. CFP excitation was set at 436/20 and FRET emission was set at 535/30. The camera exposure time was 200 ms and the interval time was 10 s. The ratio of FRET emission to CFP emission at the excitation of CFP was calculated and normalized as described before [62]. Eight to ten regions of interest in addition to a region of background were selected from one well of a 96-well plate. Three biological replications were conducted. One presentative data were presented.

**Tracer uptake in Xenopus oocytes**

Transport activities of GmSWEET10a and GmSWEET10b were detected as previously described with minor changes [20]. cRNA was prepared by T7 polymerase using mMASSAGE mMACHINE kit. Fifty nl of cRNA or water was injected into oocytes and incubated for two days. The oocyte uptake was conducted with using Na-Ringer buffer containing 100 μM sucrose (4 μCi mL^-1^ [^14^C] sucrose), glucose (4 μCi mL^-1^ [^14^C] glucose) or fructose (4 μCi mL^-1^ [^14^C] fructose). After 2 h, the oocytes were extracted by 0.1 M HNO_3_, and the radioactivity was measured by a liquid scintillation analyzer. The experiment was performed together with construct of GmSWEET15b, so that the control data of the experiment is same as previous reported [20].

**REFERENCES**

1. Bai MY, Yuan JH and Kuang HQ *et al.* Generation of a multiplex mutagenesis population via pooled CRISPR-Cas9 in soya bean. *Plant Biotechnol J* 2020; **18**: 721-31.
2. Guan YF, Huang XY and Zhu J *et al.* RUPTURED POLLEN GRAIN1, a member of the MtN3/saliva gene family, is crucial for exine pattern formation and cell integrity of microspores in *Arabidopsis*. *Plant Physiol* 2008; **147**: 852-63.
3. Danecek P, Auton A and Abecasis G *et al.* The variant call format and VCFtools. *Bioinformatics* 2011; **27**: 2156-58.
4. Gautier M, Klassmann A and Vitalis R. REHH 2.0: a reimplementation of the R package REHH to detect positive selection from haplotype structure. *Mol Ecol Resour* 2017; **17**: 78-90.
5. Jian B, Liu B and Bi Y *et al.* Validation of internal control for gene expression study in soybean by quantitative real-time PCR. *BMC Mol Biol* 2008; **9**: 59.
6. Chen MX, Wang Z and Zhu YN *et al.* The effect of TRANSPARENT TESTA2 on seed fatty acid biosynthesis and tolerance to environmental stresses during young seedling establishment in *Arabidopsis*. *Plant Physiol* 2012; **160**: 1023-36.
7. Bradstreet RB. Kjeldahl method for organic nitrogen. *Anal Chem* 1954; **26**: 185-87.
8. Wang H, Yan SJ and Xin HJ *et al.* A subsidiary cell-localized clucose transporter promotes stomatal conductance and photosynthesis. *Plant Cell* 2019; **31**: 1328-43.
9. Hou BH, Takanaga H and Grossmann G *et al.* Optical sensors for monitoring dynamic changes of intracellular metabolite levels in mammalian cells. *Nat Protoc* 2011; **6**: 1818-33.
